# Examination of Complementary Medicine for Treating Urinary Tract Infections Among Pregnant Women and Children

**DOI:** 10.3389/fphar.2022.883216

**Published:** 2022-04-27

**Authors:** Rachel E. Hudson, Kathleen M. Job, Casey L. Sayre, Lubov V. Krepkova, Catherine M. Sherwin, Elena Y. Enioutina

**Affiliations:** ^1^ Department of Pediatrics, Post-Doctoral Fellow, Division of Clinical Pharmacology, University of Utah School of Medicine, Salt Lake City, UT, United States; ^2^ Department of Pediatrics, Research Assistant Professor, Division of Clinical Pharmacology, University of Utah School of Medicine, Salt Lake City, UT, United States; ^3^ College of Pharmacy, Roseman University of Health Sciences, South Jordan, UT, United States; ^4^ Head of Toxicology Department, Center of Medicine, All-Russian Research Institute of Medicinal and Aromatic Plants (VILAR), Moscow, Russia; ^5^ Department of Pediatrics, Vice-Chair for Research, Professor, Wright State University Boonshoft School of Medicine/Dayton Children’s Hospital, Dayton, OH, United States; ^6^ Department of Pediatrics, Research Assistant Professor, Division of Clinical Pharmacology, University of Utah School of Medicine, Salt Lake City, UT, United States

**Keywords:** urinary track infection (UTI), pregnant women, children, conventional treatment, complementary medicine (CAM)

## Abstract

Urinary tract infections (UTIs) are a significant clinical problem that pregnant women and children commonly experience. *Escherichia coli* is the primary causative organism, along with several other gram-negative and gram-positive bacteria. Antimicrobial drugs are commonly prescribed to treat UTIs in these patients. Conventional treatment can range from using broad-spectrum antimicrobial drugs for empirical or prophylactic therapy or patient-tailored therapy based on urinary cultures and sensitivity to prospective antibiotics. The ongoing emergence of multi-drug resistant pathogens has raised concerns related to commonly prescribed antimicrobial drugs such as those used routinely to treat UTIs. Consequently, several natural medicines have been explored as potential complementary therapies to improve health outcomes in patients with UTIs. This review discusses the effectiveness of commonly used natural products such as cranberry juice/extracts, ascorbic acid, hyaluronic acid, probiotics, and multi-component formulations intended to treat and prevent UTIs. The combination of natural products with prescribed antimicrobial treatments and use of formulations that contained high amounts of cranberry extracts appear to be most effective in preventing recurrent UTIs (RUTIs). The incorporation of natural products like cranberry, hyaluronic acid, ascorbic acid, probiotics, Canephron^®^ N, and Cystenium II to conventional treatments of acute UTIs or as a prophylactic regimen for treatment RUTIs can benefit both pregnant women and children. Limited information is available on the safety of natural products in these patients’ populations. However, based on limited historical information, these remedies appear to be safe and well-tolerated by patients.

## 1 Introduction

Urinary tract infections (UTIs) are a common and significant clinical problem in both pregnant women and children. Infections that involve the upper urinary tract in children can result in renal scarring, hypertension, and end-stage renal disease (Shaikh et al., 2010; Salo et al., 2011). Similarly, outcomes in pregnant women can be affected by UTIs. Multiple studies investigating the outcomes associated with UTI in pregnant women have observed a positive correlation of decreased birth weight, preterm birth, and perinatal mortality (Naeye 1979; Millar and Cox 1997; Delzell and Lefevre 2000). The conventional treatment strategy for UTIs includes empiric and/or tailored antibiotic therapy. Empiric antimicrobial therapy is directed against predicted microbial agents. It usually includes broad-spectrum antibiotics and is initiated before identifying the exact causative agent and its sensitivity to antibiotics. The tailored antimicrobial therapy identifies the offending organisms through urine specimen collection and culture (Calhoun et al., 2022). Following identification, selected antibiotics are tested for efficacy or resistance on the cultured organisms. Multiple antibiotics have shown efficacy in resolving UTIs in pregnant women with a low incidence of complications (Vazquez and Abalos 2011). The decision to treat UTI in children with antibiotic therapy is based upon a risk assessment that includes several clinical and demographic risk factors that influence the probability of diagnosis (Shaikh et al., 2007). In addition, most children experience resolution of symptoms within 24 h; however, no difference in complication rates was observed in those who remained symptomatic up to 48 h after initiation of antibiotic therapy (Bachur 2000).

UTIs are commonly divided into two categories. The first is acute cystitis, involving the lower urinary tract. The second is acute pyelonephritis, which affects the upper urinary tract. Asymptomatic bacteriuria, the presence of certain bacteria levels in the urine that does not cause symptoms, is another condition that should be monitored, especially in pregnant women. Asymptomatic bacteriuria occurs in 1.9–9.5% of pregnant women (Nicolle 2003; Nicolle et al., 2019). This is significant due to the observation that asymptomatic bacteriuria in pregnant women increases the risk of developing a symptomatic UTI in 20–35% of individuals (Smaill and Vazquez 2019). Acute pyelonephritis occurs in 0.5–2% of pregnant women (Hill et al., 2005), with acute cystitis occurring at a percentage of 1–2 (Harris and Gilstrap 1981; Gilstrap and Ramin 2001). Diagnosis of asymptotic bacteriuria is defined as bacteria in the urine with no other symptoms characterizing the UTI. Specific diagnostic criteria include two consecutive urine samples where the same bacterial strain is isolated in amounts ≥10^5^ colony-forming units per milliliter (CFU/ml) (Nicolle et al., 2019). Additionally, if the urine sample is acquired through catheterization, single isolation of a bacterial strain with a quantitative count ≥10^2^ CFU/ml is considered diagnostic. Diagnosis of acute cystitis is also associated with the isolation of bacterial strains; however, specific quantities have not been delineated through studies in pregnant women. Some studies have concluded that relatively low quantities of bacteria in the urine of non-pregnant women with symptoms consistent with UTIs can serve as diagnostic signs of acute cystitis (Kunin et al., 1993). For this reason, many clinicians consider ≥10^3^ CFU/ml in a urine sample together with symptoms to be diagnostic of acute cystitis (Hooton and Gupta 2022). Typical symptoms of acute cystitis include painful urination, increased urinary frequency, and urgency (Hooton and Gupta 2022).

Fever, flank pain, nausea, and vomiting are typical symptoms of infection of the upper urinary tract or acute pyelonephritis. Pus in the urine is also a common finding of this type of UTI. Detection of a bacterial isolate in the urine, coupled with these symptoms, confirms the diagnosis (Hooton and Gupta 2022). The causative organisms isolated in pregnant women’s food are typically the same as those isolated in non-pregnant women. In around 90% of cases, *Escherichia coli* is the causative organism, with *Proteus mirabilis* and *Klebsiella pneumoniae* being somewhat common (Le et al., 2004). In addition to the specific type of organism, its virulence influences the progression and severity of infection. For example, some organisms have pili or fimbriae that can attach to surfaces more tightly. This adherence characteristic, coupled with the strength of the host immune response, is deterministic of acute pyelonephritis in women (Stenqvist et al., 1987; Svanborg and Godaly 1997). The condition of pregnancy itself does not seem to affect the virulence of *E. coli* that causes acute pyelonephritis (Stenqvist et al., 1987; Svanborg and Godaly 1997).

Pregnant women are predisposed to UTIs as a consequence of urinary tract changes that occur throughout gestation. Compression of the ureters from the gravid uterus can cause ureteral dilation, known as “hydronephrosis of pregnancy,” and hormonal effects of increased progesterone can result in smooth muscle relaxation to increase vesicoureteral reflux (LeFevre 2000; Loh and Sivalingam 2007; Habak and Griggs 2022). In addition, increased plasma volume throughout gestation can lead to decreased urine concentration and increased bladder volume (LeFevre 2000; Loh and Sivalingam 2007). Moreover, because pregnancy results in a relatively constant state of immunocompromise, the frequency of UTIs and RUTIs gain potential to increase during gestation compared to non-pregnant individuals (Habak and Griggs 2022). According to the Center for Disease Control and Prevention (CDC), diagnoses of pregnancy UTIs are most prevalent during the first trimester of pregnancy and least common during the third trimester (Ailes et al., 2018). Treatment of UTIs during pregnancy is of particular importance to avoid negative fetal effects and preterm labor. Endotoxin release from bacteria may lead to anemia and may also cause uterine contractions that can cause patients to undergo preterm labor (Habak and Griggs 2022). Of particular concern is the occurrence of RUTIs in pregnant women that cause a persistent infection that may ultimately lead to urinary obstruction or renal abscesses (Habak and Griggs 2022).

In children, urinary tract infections can also present as acute cystitis and pyelonephritis. However, the two types of UTI can be challenging to distinguish in children based on symptomology alone (Hoberman et al., 2003). This is partly because fever is present in acute cystitis and pyelonephritis cases, especially in younger children. For this reason, the two types of UTI are often not differentiated when discussing prevalence in children (Shaikh and Hoberman 2022). The prevalence of UTI in children less than 2 years of age with fever has been assessed in several studies. Hoberman, et al. found that fever is a common sign of UTIs in children less than 2 years of age. Of note was the observation that infants with no apparent source of fever (otitis media, upper respiratory infection) had twice the risk of finding UTI as the source than for infants who presented with a possible source of fever (Hoberman et al., 1993). Shaw’s group confirmed the UTI as a potential source of fever in infants with a fever that occurred with no other apparent source (Shaw et al., 1998). However, the study results also noted that another obvious source of fever was not reliable in excluding the possibility of UTI as a source (Shaw et al., 1998). A 2008 meta-analysis was instrumental in elucidating the overall prevalence of UTIs in this population (Shaikh et al., 2008). In infants presenting with fever, the incidence of UTI is around 7%.

Variability of UTI incidence in children is seen based on sex, age, and circumcision status. The highest prevalence is seen in uncircumcised boys less than 3 months of age (Shaikh et al., 2008). In the meta-analysis conducted by Shaikh et al., it was found that circumcised boys have a two to four-fold decreased risk of UTI as the source for fever compared to girls (Shaikh et al., 2008). Other significant risk factors for UTI during childhood include bladder bowel dysfunction and congenital anomalies of the kidneys and the urinary tract (Conway et al., 2007; Shaikh et al., 2010; Mattoo et al., 2021). In children under 19 years of age and older than 2 years of age, the overall prevalence was 7.8% (Shaikh et al., 2008). The clinical presentation in children younger than 2 is often non-specific, with routine irritability and fever (Zorc et al., 2005a). High fever (≥39°C (102.2°F)) and long duration (>24 h) do seem to be more indicative of UTIs (Shaw et al., 1998; Zorc et al., 2005b; Marquez and Palazzi 2016). Furthermore, the likelihood of a RUTI in the first 6–12 months after initial UTI diagnosis can range up to 30% in children (Conway et al., 2007; Peters et al., 2010). Children are most likely predisposed to RUTIs due to urinary malformation, urine stasis, and adherence of bacteria to the uroepithelial mucosa (Habib 2012). Like adults, *E. coli* is the most common causative organism for UTIs in children, causing around 80% of cases (Edlin et al., 2013). In the gram-negative classification, other organisms, such as *Klebsiella*, *Proteus*, *Enterobacter*, and *Citrobacter*, and several gram-positive species, including *Staphylococcus saprophyticus*, *Enterococcus*, and *Staphylococcus aureus,* may also be causative but are less common. As noted in adults, pili, or fimbriae, affect the virulence of *E. coli* in UTIs in children. In cases where *E. coli* is seen to have pili, a more severe response to the infection is seen in children. Higher fevers of a longer duration, a more rapid onset of symptoms, and higher white blood cell counts are clinical features in cases where pilus-positive organisms were detected (Hanson 1982).

Given that antibiotic therapy is the mainstay for treating UTIs and preventing RUTIs in these special populations, the safety and tolerability data for many antibiotics is sparse and available only for a handful of older, more established products (Bookstaver et al., 2015). In addition, the current state of antimicrobial resistance patterns for treating UTIs in pregnant women and children requires attention for possible treatment alternatives (Kalinderi et al., 2018; Kaufman et al., 2019; Asmat et al., 2021; Shrief et al., 2022). Modern medical research ethical standards often exclude these two vulnerable populations in the efficacy and safety testing of many recently developed antibiotics. This has led to significant interest in searching for viable non-drug measures for treating UTIs in pregnant women and children (Ghouri et al., 2018). This manuscript presents the current exploration of natural therapies used for the treatment and prophylaxis of UTIs among pregnant women and pediatric patients. Among many natural therapies available, cranberry, hyaluronic acid (HA), ascorbic acid, and probiotics were most investigated in clinical trials for these special populations. Additionally, we will discuss multi-component formulation containing a mixture of natural ingredients, the effectiveness of which has been demonstrated in pregnant women and children.

## 2 Conventional Treatment of Urinary Tract Infections in Pregnant Women and Children

**FIGURE 1:**
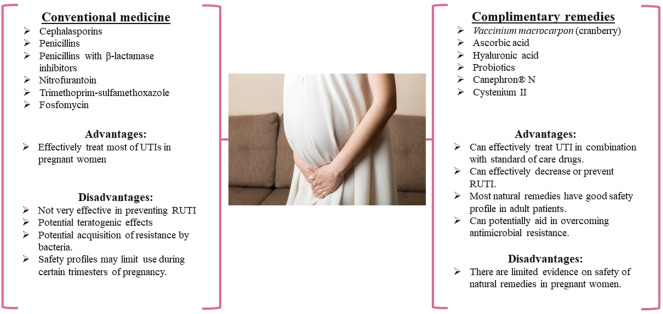
The use of conventional and complementary medicines for the treatment of UTI in pregnant women, treatments available in pregnant women.

**FIGURE 2:**
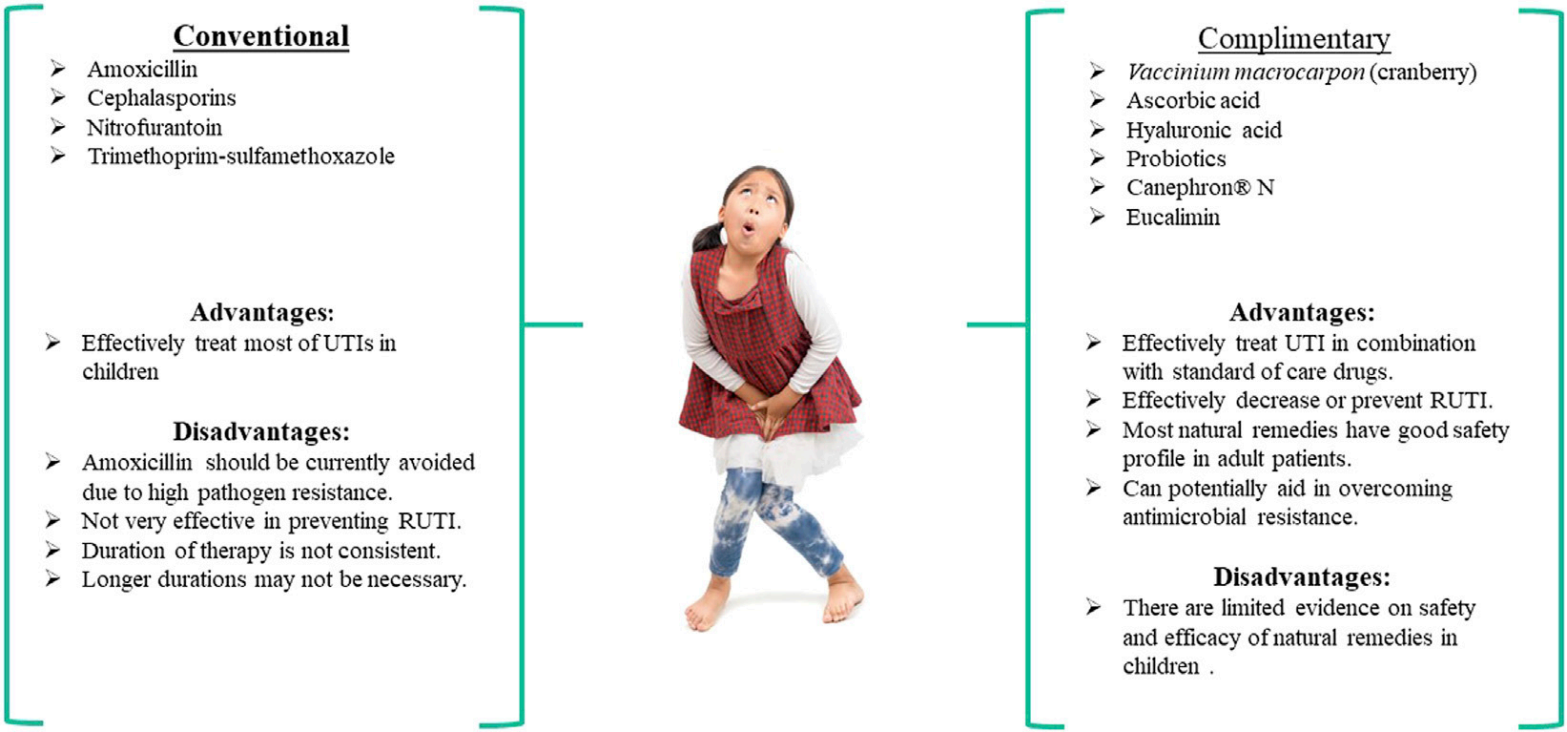
The use of conventional and complementary medicines for treating UTI in children, treatments available in children

The prescription of antimicrobial drugs is a common practice to treat UTIs in pregnant women and children (Figures 1,2). These antimicrobial drugs can be broad-spectrum antibiotics used for empiric therapy and prophylaxis for recurrent UTIs (RUTIs) or tailored to patient-specific infections.

### 2.1 Conventional Treatment of Urinary Tract Infections in Pregnant Women

UTIs are common in pregnant women. Approximately 2–15% of pregnant women will experience asymptomatic bacteriuria or symptomatic UTIs (Rac et al., 2019; Smaill and Vazquez 2019). Untreated asymptomatic and symptomatic UTIs are associated with adverse outcomes for both mother and baby (Azami et al., 2019). This includes pyelonephritis, which can progress to sepsis, necrotizing infection, and acute renal failure in pregnant women (Farkash et al., 2012; Belyayeva and Jeong 2022). Pyelonephritis occurs in up to 30% of untreated cases of asymptomatic bacteriuria (Smaill and Vazquez 2019). For the baby, adverse outcomes include intrauterine inflammation and infection, premature rupture of membranes and delivery, and postpartum infection (Ailes et al., 2018). Appropriate identification, treatment, and management of UTIs in pregnant women are integral to minimizing these outcomes.

*E. coli* is the most common infecting organism in UTIs (up to 82%) during pregnancy. Other frequent bacteria include *Klebsiella*, *Enterobacter*, *Citrobacter*, *Proteus*, *Pseudomonas*, group B *Streptococcus* (GBS), and *Enterococcus* spp. (Rac et al., 2019; Denoble et al., 2021). GBS (*Streptococcus agalactiae*) is present in 2–10% of all asymptomatic bacteriuria and has been associated with significantly elevated rates of premature rupture of membranes, preterm labor, and neonatal infection (Matuszkiewicz-Rowinska et al., 2015; Szweda and Jozwik 2016; Abou Heidar et al., 2019). Therefore, criteria for GBS infection may have lower clinical criteria for infection determination than other microbes (Perez-Moreno et al., 2017).

#### 2.1.1 Standard of Care for Urinary Tract Infections in Pregnant Women

Antibiotic regimens are the current standard of care treatment for acute and RUTIs, including asymptomatic bacteriuria, in pregnant women (Nicolle 2015; Nicolle et al., 2019; Smaill and Vazquez 2019). The choice of antibiotic regimen depends on gestational age, the target pathogen, pathogen resistance, the recurrence of infection, and clinical judgment (Tamma et al., 2017). Additional considerations include the increased risk of passing on resistant bacteria to a neonate and the potential teratogenic effects of antimicrobial treatment on the fetus (Ghouri et al., 2019). Some of the most common antibiotics include cephalosporins (first, second, and third-generation), penicillins, β-lactam antibiotics in combination with β-lactamase inhibitors, and nitrofurantoin (Rac et al., 2019). The recommended therapies for asymptomatic bacteriuria in pregnant women include nitrofurantoin after the first trimester and β-lactam antibiotics (e.g., cephalexin and ampicillin) given over a 4–7-days course (Gupta et al., 2011; Nicolle et al., 2019). These antimicrobial drugs are preferred because of their safety profile in pregnant women (Nicolle et al., 2019). However, nitrofurantoin is not recommended during the first trimester due to associated congenital disabilities. The treatment of symptomatic bacteriuria/cystitis is similar to asymptomatic bacteriuria; however, nitrofurantoin and β-lactam antibiotics (e.g., cephalexin and ampicillin) demonstrate less short-course effectiveness for acute cystitis (Gupta et al., 2011; Nicolle et al., 2019). The Infectious Diseases Society of America (IDSA) currently has no recommendations regarding further periodic screening after the antibiotic treatment course (Nicolle et al., 2019).

For example, other clinically prescribed antimicrobial drugs include amoxicillin, amoxicillin-clavulanate, and trimethoprim-sulfamethoxazole (TMP-SMX) after the first trimester through early to mid-second trimester (Vazquez and Abalos 2011; Nicolle et al., 2019; Habak and Griggs 2022). Fosfomycin administered as a single dose is also a potential therapy (Rac et al., 2019). First-line oral antimicrobial drugs for acute pyelonephritis include β-lactam antibiotics. Cephalexin and ampicillin are preferred because of the known safety profiles. Nitrofurantoin and fosfomycin are ineffective in treating pyelonephritis (Rac et al., 2019). Third-generation cephalosporins (e.g., ceftriaxone) may be recommended. When urine culture is available, first-generation cephalosporins and penicillins (e.g., ampicillin and ampicillin-sulbactam) may be recommended (Denoble et al., 2021). For severe pyelonephritis cases, carbapenems may be prescribed. For life-threatening infections, aminoglycosides may be prescribed. There are no consistent recommendations for the duration, but they are currently in the range of 10–14 days (Matuszkiewicz-Rowinska et al., 2015).

Generally, acute mild to moderate pyelonephritis is initially treated empirically based on resistance patterns and previous patient antimicrobial exposure until susceptibility testing is available (Nicolle et al., 2005; Jolley and Wing 2010; Vazquez and Abalos 2011; Matuszkiewicz-Rowinska et al., 2015; Rac et al., 2019). Parenteral administration of fluids and antibiotics should occur while the patient is febrile (≥48 h), after which the patient transitions to oral antimicrobial drugs for an extended period. In the first 48 h, ceftriaxone, cefepime, amoxicillin/clavulanic acid, and aztreonam are typically prescribed in less severe cases (Nicolle et al., 2005; Matuszkiewicz-Rowinska et al., 2015; Rac et al., 2019). For more severe cases, penicillin combinations (e.g., ticarcillin with clavulanic acid, piperacillin with tazobactam) and carbapenems (e.g., meropenem, ertapenem, doripenem) are more common (Nicolle et al., 2005; Matuszkiewicz-Rowinska et al., 2015; Rac et al., 2019).

Approximately 6–8% of pregnant women will have recurrent pyelonephritis. In these cases, prophylactic treatment (e.g., nitrofurantoin, cephalexin) may be initiated with typical treatment reinitiated when bacteriuria is detected (Matuszkiewicz-Rowinska et al., 2015). In addition, intrapartum prophylaxis is recommended by the CDC in women testing positive for GBS infection. GBS infection is associated with significant morbidity and mortality in infants (Verani et al., 2010).

#### 2.1.2 Antimicrobial Resistance Regarding Urinary Tract Infection Treatment in Pregnant Women

The rapid development of antimicrobial resistance is considered a serious threat to individual health and life expectancy (Solomon and Oliver 2014). In 2019, it was estimated that microbial resistance resulted in over 2.8 million infections and over 35,000 deaths per year in the United States alone (CDC, 2019). Resistance development is particularly concerning for both mother and infant outcomes; however, there are limited studies in pregnant women for both gram-positive and gram-negative bacteria. According to a 2019 CDC report (CDC, 2019), gram-positive GBS is present in one-fourth of pregnant women and often treated with penicillin before delivery to prevent transmission to the baby. Clindamycin and erythromycin can be used in women allergic to penicillin; however, over 40% of GBS infections are resistant to clindamycin, and over 50% are resistant to erythromycin (CDC, 2019). A recent retrospective cohort study of 334 women with gram-negative bacteriuria found that antibiotic-resistant organisms were most often (>50%) responsible for bacteriuria in pregnant women; these women were 2–3x as likely to develop pyelonephritis (Denoble et al., 2021).

Minimizing the development of microbial resistance to antimicrobial agents is the foundation for stewardship programs. Part of stewardship programs includes tracking local or regional resistance patterns. These patterns often vary. For example, Chelkeba et al. (Chelkeba et al., 2022) recently performed a systematic review of antimicrobial resistance patterns in Ethiopia. In pregnant women with bacteriuria, 83% of *E. coli* were multidrug-resistant. Kaye et al. (Kaye et al., 2021) performed a retrospective study of 1.5 million *Escherichia coli* isolates from the United States. They found that 14.36% of *E. coli* isolates in urine samples in adult and adolescent females showed a non-susceptible phenotype to multiple drugs. Resistance and development of resistance to specific drugs showed significant variation throughout regions, states, and counties. For example, observations showed that the New England region had an average increase in multidrug resistance of 7.0% (6.5–7.5), whereas the East South-Central region had an average increase of 16.7% (16.1–17.3). Understanding these patterns and trends is vital to maximizing mother and baby outcomes as a recurrent infection is common in pregnant women (Schneeberger et al., 2015).

Resistance to antimicrobial drugs often occurs rapidly. For example, *E. coli* showed resistance to cefotaxime, an extended-spectrum beta-lactam, 3 years after it was introduced (CDC, 2019). Kim et al. recently studied 265 infants (<1 year of age) and found that antibiotic use by mothers during pregnancy was associated with isolation of the extended-spectrum beta-lactamase (ESBL)-producing bacteria from specimens of infants with UTIs (Kim et al., 2022). Currently, there are very few therapeutic options available for the treatment of ESBL-positive infections, mainly due to ESBL-producing bacterium ability to hydrolyze third-generation cephalosporins (Pana and Zaoutis 2018). Carbapenem treatment has been set as the preferred treatment for infections with ESBL-producing bacteria (Pana and Zaoutis 2018). Fosfomycin has been recommended specifically for the treatment of UTIs with ESBL-producing bacteria. Efforts are being made to develop new antimicrobial drugs; however, the development of antimicrobial drugs is being outpaced by the development of resistance (CDC, 2019).

Antimicrobial regimens are critical components for treating symptomatic UTIs; however, the benefits of using antimicrobial agents to treat uncomplicated UTI and asymptomatic bacteriuria are not entirely understood. In general, the need to treat asymptomatic UTIs is considered questionable. Current issues associated with antimicrobial use include patient compliance, the development of resistance, potential adverse effects for both mother and baby, and the overall treatment effectiveness. Additionally, current testing methods of UTIs appear to have low sensitivity and specificity for screening bacteriuria, leukocyte esterase, and nitrite (Jido 2014; Gehani et al., 2019). These issues complicate the development of effective antimicrobial treatments for UTI and RUTI treatments. Additional therapies with the potential to reduce infection and exposure to antimicrobial drugs should be explored.

### 2.2 Conventional Treatment of Urinary Tract Infections in Children

UTI serves as one of the most common bacterial infections during childhood, such that 6–8% of children with urinary symptoms will be diagnosed with a UTI (Shaikh et al., 2008; O’Brien et al., 2013; Kaufman et al., 2019). The treatment of childhood UTIs should be recognized and treated during the early phase of the disease to provide rapid recovery from complaints, prevent related complications, and deter permanent renal parenchymal damage (Beetz and Westenfelder 2011). Prescribed antimicrobial regimens are the current standard of treatment for acute and RUTIs in children. The causative bacterial pattern determines antimicrobial therapy and prophylaxis in pediatric patients. The most common causative organism in childhood UTIs is *E. coli* (White 2011; Edlin et al., 2013), followed by *Klebsiella, Proteus, Enterobacter, Citrobacter, Staphylococcus saprophyticus,* and *Enterococcus* (Shaikh et al., 2007; White 2011).

Prescribed conventional drugs should be well tolerated by infants and children, highly effective against usual invading bacteria, and should be guided by regional bacteria resistant patterns such that only a minimal proportion of organisms are resistant (Beetz and Westenfelder 2011; Mattoo et al., 2021). However, empiric therapy with a broad-spectrum antibiotic is reasonable until culture results are available if there is a high clinical suspicion of a UTI (Alper and Curry, 2005; Roberts, 2011). Oral antibiotics should be given immediately in all toxic-appearing children who are pale, lethargic, or inconsolably irritable and in nontoxic children with a probable UTI, whereas other cases can delay antibiotic treatment until results from a urine culture are available (Luszczak 2001; Porter and Kaplan 2011; Balighian and Burke 2018).

#### 2.2.1 Standard of Care for Urinary Tract Infections in Children

Traditional first-line antibiotic treatment of UTIs in young children has typically included oral amoxicillin, a sulfonamide-containing antimicrobial, or a cephalosporin (Beetz and Westenfelder 2011; Porter and Kaplan 2011; Subcommittee on Urinary Tract Infection et al., 2011; White 2011; Balighian and Burke 2018). The antibiotic of choice should ideally be patient-specific and tailored to individual bacterial susceptibility patterns (Nickavar and Sotoudeh 2011; Balighian and Burke 2018; Kaufman et al., 2019). Currently, the vast majority of bacteria that cause UTIs are sensitive to third-generation cephalosporins (Shaikh et al., 2016b; Mattoo et al., 2021). A first-generation cephalosporin, TMP-SMX, or nitrofurantoin may be a more reasonable choice of antibiotic in non-febrile pediatric patients diagnosed with a UTI (Shaikh et al., 2016b; Mattoo et al., 2021). Amoxicillin should be currently avoided, as most bacteria are resistant to this antibiotic (Mattoo et al., 2021).

The reported duration of oral antibiotic treatment can vary between a shorter duration (6–9 days) and a longer duration (>10 days). A review carried out from the Cochrane Database that analyzed 2–4 days (short duration) versus 7–14 days (standard duration) of oral antibiotic treatment among children diagnosed with lower UTIs found no differences in positive urine culture samples between therapy durations immediately following treatment (Michael et al., 2003). In addition, no significant differences existed between the short and standard treatment durations for the development of resistant organisms (Michael et al., 2003). Nonetheless, despite comparable outcomes observed for shorter and longer durations of treatment, the typical length of oral antibiotics prescribed to pediatric patients diagnosed with a UTI is 14 days (Michael et al., 2003; Fitzgerald et al., 2012; Fusco et al., 2020; Mattoo et al., 2021).

While the majority of children with a diagnosed UTI can be treated with an oral antibiotic (Hoberman et al., 1999; Bloomfield et al., 2005; Hodson et al., 2007), patients unable to receive oral medication should be administered an antimicrobial agent via the parenteral route (Subcommittee on Urinary Tract Infection et al., 2011). Parenteral administration should continue until the patient exhibits clinical improvement and can retain oral fluids and medications (Subcommittee on Urinary Tract Infection et al., 2011; Balighian and Burke 2018). Both oral and parenteral antibiotic therapy appears to have similar effective rates for UTIs among children (Hoberman et al., 1999; Hodson et al., 2007). Antibiotic treatment should be started within the first 48 h of fever onset in children with a febrile UTI to decrease the risk of renal scaring (Oh et al., 2012; Shaikh et al., 2016c; Karavanaki et al., 2017; Mattoo et al., 2021) with an expectant resolution of fever in 68% of patients by 24 h and 92% by 72 h (Bachur 2000).

Parenteral antibiotic treatment durations show a similar trend. A recent study from 2019 reported that ≤7 days of parenteral antibiotic therapy in infants older than 60 days diagnosed with a bacteremia UTI appears to be as safe and effective as the conventional, longer-duration treatment (Desai et al., 2019). Overall, oral antibiotics with low resistance patterns for 7–10 days for upper UTI and 3 days for lower UTI are recommended for pediatric patients older than 3 months (Baumer and Jones 2007). In addition, the regimen 2–4 days of parenteral antibiotics followed by a switch to oral antibiotics for 10 days has been recommended for vomiting patients unable to receive oral antibiotics (Baumer and Jones 2007). Following treatment and resolution of UTI symptoms, follow-up urinalysis and cultures are not required unless clinically indicated (Currie et al., 2003; Oreskovic and Sembrano 2007; Mattoo et al., 2021).

In addition to first-line antibiotic treatment, preventive treatment with antibiotic prophylaxis is a commonly used practice to deter RUTIs in patients who experience two or more infections during a 6-month period (Nickavar and Sotoudeh 2011; Kaufman et al., 2019). Among children diagnosed with a UTI, over 30% will undergo recurrent infection, which supports prophylactic antimicrobial administration (Keren et al., 2015; Kaufman et al., 2019). Several studies have explored the effectiveness of antibiotic prophylaxis for preventing UTI recurrence among children; however, results remain unclear and contradictory. Nitrofurantoin and TMP-SMX are the most common antibiotics used for UTI prophylaxis in children, and the long-term use of these low-dose antibiotics are mostly safe with minimal related adverse effects (Coraggio et al., 1989; Uhari et al., 1996; Song and Kim 2008). Whereas the Randomized Intervention for Children with Vesicoureteral Reflux (RIVUR), Prevention of Recurrent Urinary Tract Infection in Children with Vesicoureteral Reflux and Normal Renal Tracts (PRIVENT), and Careful Urinary Tract Infection Evaluation (CUTIE) trials suggest fewer infections in children receiving prophylactic antibiotics with significant benefits in children diagnosed with vesicoureteral reflux (VUR) and bladder-bowel dysfunction (Craig et al., 2009; Investigators et al., 2014; Shaikh et al., 2016a), majority of trials have shown no beneficial effect of antibiotic prophylaxis (Garin et al., 2006; Montini et al., 2008; Pennesi et al., 2008; Roussey-Kesler et al., 2008). A trial exploring prophylactic administration of TMP-SMX showed that children had a 3-times higher chance of recurrent infections than children who received a placebo (Hari et al., 2015). From these results, it may be plausible to conclude that young children with high-grade VUR are likely to benefit most from antibiotic prophylaxis (Uhari et al., 1996). Variations in trial results could be attributable to differences in patient age and inclusion and exclusion criteria. Nonetheless, prophylactic prevention practices remain controversial as they have been shown to contribute significantly to antibiotic resistance from increased dosing and exhibit inconsistent results (Conway et al., 2007; Beetz and Westenfelder 2011; Nickavar and Sotoudeh 2011; Porter and Kaplan 2011; White 2011; Balighian and Burke 2018; Kaufman et al., 2019).

#### 2.2.2 Antimicrobial Resistance Regarding Urinary Tract Infection Treatment in Children

The efficacy of commonly prescribed antibiotics to treat UTIs in children has been negatively altered in recent years due to increased pathogen resistance rates. Inappropriate antibiotic treatment and dose selection can result in treatment failure and contributes to increased resistance (Samanci et al., 2020). Specifically, the broad use of antibiotics and low-dose prophylactic antibiotic use has been blamed for the development of resistance. While there are regional differences, pathogenic resistance rates to antibiotics chosen for UTI treatment in children exhibit a steady increase worldwide (Pena et al., 1995; Ladhani and Gransden 2003; Prais et al., 2003; Lutter et al., 2005; Budnik and Bevzenko 2020; Samanci et al., 2020). Within the United States, amoxicillin and TMP-SMX have traditionally been chosen as first-line antibiotics for UTI in children; however, increased rates of *E. coli* resistance have made these antibiotics less ideal (Lutter et al., 2005; White 2011; Samanci et al., 2020). A recent study investigating the prevalence of antibiotic resistance among children with UTI over the past 10 years, specifically the sensitivity of *E. coli* to commonly prescribed antibiotics, revealed alarming increases in resistance rates since 2009 (Budnik and Bevzenko 2020). Specifically, ampicillin and amoxicillin showed a low sensitivity level (3.5 ± 32.14%) (Budnik and Bevzenko 2020). Common oral therapy options incorporate sulfisoxazole, ciprofloxacin, and cephalosporins, including ceftazidime, cefixime, cefpodoxime, cefprozil, cephalexin (Alper and Curry 2005; Lutter et al., 2005; Beetz and Westenfelder 2011; Nguyen and Whitehill 2011; Porter and Kaplan 2011; White 2011; Budnik and Bevzenko 2020). Among these, ceftazidime and ciprofloxacin have shown a relatively high sensitivity level, 77.4 ± 3.34% and 83 ± 2.81%; however, the use of these antibiotics have subsequently caused a rapid increase in *E. coli* resistance rates such that they have almost doubled over the past 5 years (Budnik and Bevzenko 2020). Due to the developing resistance of urinary tract pathogens to antibiotics, it may be advantageous to explore complementary medicine options among children who experience RUTIs to improve patient outcomes.

## 3 Complementary Medicine for Treating Urinary Tract Infections in Pregnant Women and Children

The exploration and use of complementary therapeutic options for UTIs in pregnant women and pediatric patients have gained popularity to combat pathogen resistance rates associated with antimicrobial use (Figures 1, 2). In addition, the use of natural therapies as complementary medicine is relatively safe and well-tolerated in these special populations, which supports their administration to combat RUTIs. Table 1 outlines the natural therapies and associated clinical trials and multi-component formulations that have been used for clinical application.

**TABLE 1:** Summary of complementary therapeutic options for treating and preventing urinary tract infections in pregnant women and children.

Complimentary Therapeutic	Ingredients	Mechanism of Action	Dosage Forms	Route of Administration	Applications
*Vaccinium macrocarpon* (cranberry)	cranberry	Inhibits adhesion of UTI-causing bacteria to uroepithelial cells	tablet, juice, cocktail, capsule	oral	Pregnant women: Prophylactic UTI prevention (Wing et al., 2008
					Essadi and Elmehashi, 2010
					Wing et al., 2010
					Wing et al. (2015)
					Children: Prophylactic UTI prevention (Ferrara et al., 2009
					Afshar et al., 2012
					Mutlu and Ekinci, 2012
					Salo et al., 2012
					Fernández-Puentes et al., 2015
					Wan et al. (2016)
Hyaluronic Acid	hyaluronic acid, Deflux^®^ (dextranomer and hyaluronic acid)	Contributes to bladder epithelium glycosaminoglycan layer and prevents *E. coli* from infecting urothelial cells	prefilled syringe	intravesical	Pregnant women: Prophylactic UTI prevention (Arguelles Rojas et al., 2021)
					Children: Prophylactic UTI prevention (Traxel et al., 2009
					Fidan et al., 2015
					Ure et al., 2016
					Cicek et al. (2020)
Ascorbic Acid (vitamin C)	vitamin C	Potentially acidifies the urine to decrease the risk of oxalate formation and provides bactericidal effects from nitrate reduction to nitric oxide	not specified	oral	Pregnant women: Prophylactic UTI prevention (Ochoa-Brust et al., 2007)
					Children: Prophylactic UTI symptom relief (Yousefichaijan et al., 2018)
Probiotics	*Lactobacillus* strains, *Bifidobacterium* strains, and *S. boulardii* bacteria	Immune system modulation for anti-inflammatory effects and promote the production of antimicrobial properties (H_2_O_2_, lactic acid, and broad-spectrum antibiotics)	capsule, suppository	oral	Pregnant women: Prophylactic UTI prevention (Ho et al., 2016; Gutierrez 2017; Liu et al., 2020)
					Children: Prophylactic UTI prevention (Lee et al., 2007
					Mohseni et al., 2013
					Madden-Fuentes et al., 2015
					Lee et al., 2016
					Sadeghi-bojd et al., 2019
					Daniel et al. (2020)
Canephron^®^ N	centaury powder, lovage roots, and rosemary leaves	Diuretic, anti-nociceptive, anti-spasmodic- and anti-adhesive effects	tablet	oral	Pregnant women: Prophylactic UTI prevention and potential for UTI treatment (Potapo et al., 2004
					Ordzhonikidze et al., 2009
					Medved et al., 2015
					Lokshin, (2018)
					Children: Prophylactic UTI prevention (Sukalo et al., 2004
					Kirillov et al., 2007
					Voznesenskaya and Kutafina, 2007
					Dilin et al. (2008)
Cystenium II	cranberry, D-mannose, and vitamin C	Inhibits adhesion of UTI-causing bacteria to uroepithelial cells and possible immunomodulation of regulatory T cell differentiation	tablet	oral	Pregnant women: Prophylactic UTI prevention (Nashivochnikova and Leanovich, 2020)
					Children: Clinical investigation needs to be conducted

### 3.1 *Vaccinium macrocarpon* (Cranberry)

American cranberry (*Vaccinium macrocarpon*) has a complex composition of bioactive components, including proanthocyanidins (falcan-3-ols, A-type procyanidins, anthocyanins, benzoic acid, and ursolic acid), anthocyanins, phenolic acids (hydroxybenzoic and hydroxycinnamic acids), terpenes, and flavonols (Blumberg et al., 2013). Proanthocyanidins, specifically A-type procyanidins, highly influence the biological activity of cranberry against UTIs. This component has been shown to aid in the inhibition of adhesion of *E. coli* and other gram-negative bacteria to uroepithelial cells, which predominately serve as the initial step of a UTI (Schmidt and Sobota 1988; Lynch 2004; Howell et al., 2005; Blumberg et al., 2013). Specifically, cranberry has been shown to inhibit hemagglutination of *E. coli* by preventing the adhesion of type 1 and P-fimbriated pathogens to the uroepithelium (Zafriri et al., 1989; Howell et al., 1998; Lynch 2004; Guay 2009). It has been hypothesized that this effect happens in the gut rather than the urinary tract (Salo et al., 2012). Cranberry residues present in stool potentially induce a shift toward a less uropathogenic bacterial flora and subsequently result in prolonged protection against UTI (Salo et al., 2012). While some studies have concluded cranberry supplementation does not alter the frequency of bacteria in bladder urine or fatty acid composition of stools among pediatric patients, an analysis on bacteria function in pregnant women has yet to be evaluated (Schlager et al., 1999; Kontiokari et al., 2005). Nonetheless, before introducing clinical antibiotics, cranberry was traditionally used as the popular treatment for UTIs (Robbers and Tyler 1999).

#### 3.1.1 Effectiveness of Cranberry Formulations for Urinary Tract Infection Treatment in Pregnant Women

Women experience higher UTI incidence during pregnancy. During pregnancy, the urinary tract changes, including ureteral dilation and hormonal effects, predisposing pregnant women to infection (Habak and Griggs 2019). Ingestion of cranberry during pregnancy is considered safe, and there is scientific evidence that cranberry is of minimal risk to pregnant mothers and the fetus (Dugoua et al., 2008). A survey that included 400 pregnant women detected no adverse events when regularly consuming cranberry (Dugoua et al., 2008). This is particularly important as there are limited therapeutic options available to treat UTIs during pregnancy. A few clinical trials have evaluated the effectiveness of cranberry against UTI in pregnant women. Among these, a pilot study that evaluated the compliance of cranberry capsules during pregnancy showed that consumption of cranberry capsules was tolerable in pregnant women with an 82% compliance rate over 6 months (Wing et al., 2015). An additional randomized control trial was carried out to compare the effectiveness of cranberry juice versus placebo in preventing asymptomatic bacteriuria associated with UTI in women under 16 weeks of pregnancy. Of the 188 pregnant women included women who received multiple cranberry doses each day had a 57% reduction in asymptomatic bacteriuria and a 41% reduction in symptomatic UTIs; however, while others have reported daily ingestion of cranberry to be tolerable for women during pregnancy, this study reported a high incidence of withdrawal (38.8%) due to gastrointestinal intolerability (Wing et al., 2008).

Nonetheless, the UTI frequency reduction rates from this trial are supported by an additional randomized controlled trial such that 70.5% of pregnant participants who consumed cranberry juice reported a reduction in UTI frequency compared to 32.16% of pregnant participants who consumed water (Essadi and Elmehashi 2010). In addition, a pilot study evaluating the *in vivo* cytokine profiles of urinary samples from pregnant women ingested cranberry juice reported significantly low levels of interleukin-6, a cytokine involved in the early phase of inflammation, in women who ingested cranberry multiple times a day versus the placebo (Wing et al., 2010). However, pregnant women who took cranberry juice once or twice a day did not demonstrate any differences in cytokine profiles versus the placebo. Studies in pregnant women were primarily focused on utilizing cranberry as prophylaxis to prevent RUTIs rather than treatment of primary UTI.

#### 3.1.2 Effectiveness of Cranberry Formulations for the Treatment of Urinary Tract Infection in Children

The use of cranberry has been confirmed to be safe in both infants and children (Fernández-Puentes et al., 2015). Several clinical trials that explored the effects of cranberry supplementation among pediatric patients have demonstrated reduced UTI incidence in children with recurrent episodes, both male and female (Ferrara et al., 2009; Afshar et al., 2012; Mutlu and Ekinci 2012; Salo et al., 2012; Fernández-Puentes et al., 2015; Wan et al., 2016). In a randomized placebo-controlled study that evaluated the effectiveness of cranberry juice with high concentrations of proanthocyanidins and had a mean patient age of 9.5 years, ingestion of cranberry juice reduced UTI risk by 65% (Afshar et al., 2012). An additional study that explored cranberry juice consumption in children treated for UTI exhibited that cranberry juice reduced related antimicrobial use by significantly decreasing the number of days on antimicrobial drugs from 17.6 to 11.6 days (Salo et al., 2012). In a study that evaluated the benefits of a highly concentrated cranberry juice for the prevention of RUTIs in uncircumcised boys, cranberry juice decreased the incidence of bacteria (urine cultures greater than or equal to 1 × 10^5^ were 25% for cranberry, 37% for placebo), mainly *E. coli*, and resulted in fewer RUTI episodes (Wan et al., 2016). While these studies explored and assumed daily consumption of cranberry juice versus placebo, Ferrara et al. demonstrated differences in daily consumption versus non-daily consumption of cranberry juice versus placebo. Among female children, cranberry juice resulted in fewer UTIs when ingested daily rather than 5 days a month (18.5 versus 42.3%) (Ferrara et al., 2009). In a study that observed effects of cranberry capsules instead of juice in children with neurogenic bladder (NB) caused by myelomeningocele, cranberry supplementation significantly decreased infection rate and frequency of pyuria (Mutlu and Ekinci 2012). Prophylactic comparison with a cranberry cocktail versus an antibiotic (trimethoprim) in a controlled, double-blind Phase III clinical trial demonstrated cranberry cocktail prophylaxis was not inferior to antibiotic prophylaxis (cumulative rate of UTI: 26 versus 35%) in children older than 1 year of age (Fernández-Puentes et al., 2015). However, the same study showed that the cranberry cocktail was inferior to antibiotic prophylaxis in children younger than 1 year (cumulative rate of UTI: 35 versus 28%) (Fernández-Puentes et al., 2015). This suggests that age may play a role in pediatric patient outcomes when utilizing cranberry prophylaxis for UTI prevention. An additional study that explored the effects of a cranberry cocktail prophylaxis versus placebo (water) with a cross-over design in pediatric patients presented no differences in cranberry versus placebo (Foda et al., 1995), which contradicts previous studies. These dissimilarities could be attributable to differences in age, total cranberry content consumption, and gender among studies. In general, cranberry supplementation appears to aid in RUTI prevention when administered as prophylaxis in the form of juice, capsule, or cocktail and gains potential to decrease antibiotic use in pediatric patients for overcoming antibiotic resistance rates.

### 3.2 Hyaluronic Acid

HA, also known as hyaluronan or hyaluronate, is a naturally occurring non-sulfated glycosaminoglycan (GAG) and comprises repeating β-1,4-D-glucuronic acid and β-1,3-N-acetylglucosamine units (Morales et al., 1997; Fallacara et al., 2018; Gupta et al., 2019). Due to its composition, HA has great viscoelastic nature, ability to form highly hydrated matrices, biocompatibility, and hygroscopic properties (Necas et al., 2008; Gupta et al., 2019). It serves as the main mucopolysaccharide constituent of the extracellular matrix in various tissues and is present in the majority of body fluids (Morales et al., 1997; Necas et al., 2008; Fallacara et al., 2018; Gupta et al., 2019). HA is most abundantly present in articular cartilage and synovial fluid (Necas et al., 2008; Gupta et al., 2019). Regarding urinary health, HA contributes significantly to cell proliferation and migration and, therefore, can act as a protective barrier to the urothelium (Iavazzo et al., 2007). Several properties of HA appear to contribute to its prophylactic ability in preventing recurrence of UTIs, including its capacity to inhibit the adherence of immune complexes to polymorphonuclear cells, inhibit leukocyte migration and aggregation based on the degree of viscosity, regulate cell proliferation of fibroblasts and endothelial cells, and enhance connective tissue and wound healing (Iavazzo et al., 2007; Gupta et al., 2019). Specifically, the re-establishment of the bladder epithelium GAG layer with HA may be the principal action for increased urinary tract health (Goddard and Janssen 2018). It is important to note that HA is not directly toxic to *E. coli*; however, it has been observed to provide a barrier function and prevent *E. coli* from infecting urothelial cells by coating the urothelial surface (Lee et al., 2010; Sihra et al., 2018). Altogether, evidence from the supplementation of HA in pregnant women and pediatric patients suggests HA is a valid complementary therapeutic option for preventing UTIs.

#### 3.2.1 Effectiveness of Hyaluronic Acid for Treatment of Urinary Tract Infection in Pregnant Women

Exploration of HA for complementary therapy in pregnant women has not been thoroughly explored; however, the results from a case presentation where HA treatment was given to a woman at 27.2 weeks of gestation to treat interstitial cystitis, a syndrome associated with urinary symptoms, were significantly improved following puerperium (Arguelles Rojas et al., 2021). Although a UTI does not always accompany interstitial cystitis, patients diagnosed with this syndrome have reported UTI at interstitial initiation (Held et al., 1990; Driscoll and Teichman 2001; Porru et al., 2004; Warren et al., 2008). Nonetheless, HA treatment caused partial improvement of urinary symptoms during gestation with complete eradication of symptoms 6 months after pregnancy when HA doses were continued (Arguelles Rojas et al., 2021). Following 6 months, the patient was asymptomatic. The use of HA treatment during pregnancy for UTI may also be supported by the findings from a study that evaluated the concentrations of HA in various urogenital organs of pregnant and non-pregnant rats (Laurent et al., 1995). In addition, high levels of HA content were observed in the vagina, and urinary bladder of both pregnant and non-pregnant rats, with the highest level of HA, observed in the vagina for the pregnant rats (Laurent et al., 1995). Finally, the high HA content in the urinary bladder, even observed during pregnancy, supports its protective properties in UTI treatment and prevention. Regardless, administration of HA during pregnancy for UTI treatment is warranted and needs to be more thoroughly studied in a clinical setting.

#### 3.2.2 Effectiveness of Hyaluronic Acid for Treatment of Urinary Tract Infection in Children

Pediatric patients experience a high prevalence (2–8%) of UTIs with increased risk amid patients with underlying VUR and bladder instability such as NB (Saadeh and Mattoo 2011; Fidan et al., 2015). A clinical study that evaluated the use of intravesical HA in conjunction with TMP-SMX antibiotic prophylaxis in complicated (including patients with VUR or NB) and uncomplicated pediatric males and females found that patients who received HA administration resulted in complete response with sterile urine cultures of 53% for complicated patients and 71.4% for uncomplicated patients following a 2-years monthly follow-up period (Fidan et al., 2015). In addition, 25% of complicated patients and 14.3% of uncomplicated patients reported a partial response, and RUTI frequencies were less than half compared to the period prior to HA therapy (Fidan et al., 2015). Another clinical study assessed the effectiveness and safety of intravesical HA ability to reduce RUTIs in complicated pediatric patients who had spina bifida (SB) and NB versus a control group when antibiotic prophylaxis was given (Cicek et al., 2020). The mean UTIs per patient month were significantly decreased (*p* < 0.001) for the HA-treated patients compared to the control group (Cicek et al., 2020). Both studies suggest intravesical HA as an applicable treatment option for RUTIs in pediatric patients with and without complications. An additional study assessed the frequency of postoperative UTIs following Deflux® treatment in 75 pediatric patients (mean age of 6.59 years) with primary VUR. Deflux® is an endoscopically injected drug comprised of dextranomer and hyaluronic acid. The study demonstrated a 100% success rate in patients with grade I and II VUR, 91% in patients with grade III, and 82.6% in patients with grade IV (Ure et al., 2016). A retrospective study conducted earlier investigated the effectiveness of Deflux® for the prevention of UTIs in children with VUR (Traxel et al., 2009). In this study, 13% of treated patients developed a postoperative UTI and 3.5% febrile UTI (Traxel et al., 2009). It has been determined that patients with pre-operative UTIs and bladder dysfunctions may be at risk of developing post-operative UTIs. The overall results are promising and support the administration of Deflux® as an option for HA treatment in pediatric patients with VUR to prevent UTIs.

### 3.3 Ascorbic Acid (Vitamin C)

Ascorbic acid (Vitamin C) is water-soluble and a low-molecular-weight carbohydrate that is an essential micronutrient for humans. The reduced form, ascorbate, is related to the biological role of ascorbic acid and is responsible for many enzymatic and nonenzymatic functions that result in the production of building blocks for collagen, regulation of transcription of several genes, modulation of vascular function, and action as a powerful antioxidant (Lykkesfeldt et al., 2014). Ascorbic acid has been recommended as a supplement for preventing UTIs due to evidence of its ability to acidify the urine and decrease the risk of oxalate stone formation (Masson et al., 2009; Hickling and Nitti 2013). This effect on urine pH has been controversial, as some patients who consume reasonable quantities of daily ascorbic acid (1,000 mg per day) have not resulted in significantly decreased urine pH (Beerepoot and Geerlings 2016). It has also been proposed and supported with *in vitro* data that the presence of ascorbic acid may reduce nitrite, a product that bacteria can excrete, to nitric oxide and other nitrogen-reactive intermediates that ultimately have bactericidal effects (Carlsson et al., 2001; Carlsson et al., 2005; Borran et al., 2020). Investigation of ascorbic acid as a therapeutic option in pregnant women and pediatric patients is limited, yet studies have produced promising results.

#### 3.3.1 Effectiveness of Ascorbic Acid for Treating Urinary Tract Infection in Pregnant Women

Prophylactic administration of ascorbic acid in pregnant women suggests favorable outcomes for UTI prevention. A single-blinded clinical trial compared an oral treatment with 200 mg ferrous sulfate, 5 mg folic acid, and 100 mg ascorbic acid and treatment with ferrous sulfate and folic acid only of pregnant women (Ochoa-Brust et al., 2007). The addition of ascorbic acid to the formulation appeared to result in lower overall UTIs. Specifically, the treatment group supplemented with ferrous sulfate, folic acid, and ascorbic acid had a UTI frequency of 12.7% compared to 29.1% of pregnant women who received ferrous sulfate and folic acid (*p* = 0.03). Patients who acquired a UTI during the study were given treatment according to positive urine cultures, but antibiotics were not given otherwise. This study suggests that daily intake of ascorbic acid may play a role in reducing UTIs and potentially could be used for RUTI prophylaxis.

Interestingly, three infants from the group of pregnant women who did not receive ascorbic acid had low birth weights, while no infants in the ascorbic acid-treated group were born with low birth weights. These results further support ascorbic acid supplementation in pregnant women for overall improved health of both mother and infant (Ochoa-Brust et al., 2007). Therefore, investigation of ascorbic acid as a monotherapy prophylactic agent in pregnant women is necessary and should be considered for future exploration.

#### 3.3.2 Effectiveness of Ascorbic Acid for the Treatment of Urinary Tract Infection in Children

Ascorbic acid supplementation as a possible tool to relieve UTI symptoms in children has been evaluated. One clinical trial included 152 female children who tested positive for UTI in a hospital setting and were assigned to either an ascorbic acid supplemented treatment group or a placebo control (Yousefichaijan et al., 2018). These pediatric patients were also given routine parenteral ceftriaxone for admitted patients followed by oral cefixime antibiotics following patient discharge in conjunction with ascorbic acid or placebo. The trial found that ascorbic acid supplementation resulted in controlled symptoms associated with UTIs, including fever, dysuria, urinary urgency, and dribbling urine with decreased frequency of these symptoms in the treatment group (Yousefichaijan et al., 2018). These results suggest that ascorbic acid plays a role in reducing UTI symptoms among pediatric females when given intravenous and oral antibiotics; however, additional exploration is needed to confirm these results.

### 3.4 Use of Probiotics for the Treatment of Urinary Tract Infection

Probiotics have gained attention as a complementary treatment of UTI infections in women, including pregnant women and children (Gupta et al., 2017; Meena et al., 2021). It has been believed that the urinary tract and, specifically, human urine are sterile. Lately, it has been established that men’s and women’s urinary microbiome consists of *Streptococcus, Escherichia, Enterococcus, and Citrobacter* (Perez-Carrasco et al., 2021). *Lactobacillus* is a dominant genus present in a healthy woman’s vagina. Therefore, probiotic formulations intended to treat UTIs should contain dominant microbiome bacterial strains like *Lactobacillus* spp. (Akgül and Karakan, 2018).

The proposed rationale for using probiotics was the effect of probiotics on immune system functions. Dendritic cells recognize probiotic bacteria, leading to their maturation activation and stimulating IgA production by B cells (Macpherson and Uhr 2004). Primed by *Lactobacillus rhamnosus,* dendritic cells can reduce CD4^+^ T cell proliferation and IL2, IL4, and IL10 production (Braat et al., 2004). Teichoic acid found in gram-positive bacteria, specifically in some strains of *Lactobacillus plantarum,* demonstrate anti-inflammatory properties potentially via increased production of IL10 by immune cells (Grangette et al., 2005). *Lactobacillus* strains produce antimicrobial peptides (e.g., bacteriocins), H_2_O_2_, lactic acid, and broad-spectrum antibiotics (e.g., reuterin produced by *Lactobacillus reuteri*) (Cleusix et al., 2008; Dobson et al., 2012; Gupta et al., 2017). Additionally, probiotics can inhibit the growth of *E. coli*, the primary causative agent of UTIs in women and children (Beerepoot et al., 2013). Probiotics support intestinal wall integrity (Oelschlaeger 2010; Gupta et al., 2017). Others (e.g., *Lactobacillus brevis* CD2 and *Lactobacillus salivarius* FV2) are capable of adhering to the vaginal epithelial cell walls, thus preventing the adhesion of pathogenic strains to epithelial cells (Cribby et al., 2008; Gupta et al., 2017).

Most studies investigating the effectiveness of probiotics for treating UTIs administered the probiotics via the oral route. The assumption was that probiotics administered orally could repopulate the vagina by colonizing the rectum, moving to the perineal area, and then to the vagina (Morelli et al., 2004). A few trials evaluated the effectiveness of intravaginal probiotic delivery or bladder instillation.

#### 3.4.1 Probiotic Treatment of Urinary Tract Infections in Pregnant Women

Animal studies conducted by Reid et al., in 1985 demonstrated that inserting *Lactobacillus casei* into the bladder of rats previously infected with uropathogenic bacteria could prevent the development of bladder infection in 84% of treated animals (Reid et al., 1985). The randomized placebo-controlled clinical trial conducted by Stapleton et al. found that prophylactic treatment of pre-menopausal women with RUTIs daily for 5 days and then weekly for 10 weeks with vaginal suppositories containing *Lactobacillus crispatus* resulted in a significant reduction of recurrent infections (Stapleton et al., 2011). This study determined that the high level of colonization in the vagina with *Lactobacillus crispatus* was associated with reducing RUTIs. The single intravaginal administration of *Lactobacillus rhamnosus* GR-1 to a woman with RUTIs resulted in up to 8 weeks of survival of *Lactobacillus* in the vagina (Reid et al., 1994). In addition, the treatment of bacterial vaginosis with an oral suspension of *Lactobacillus rhamnosus* GR-1 and *Lactobacillus fermentum* RC-14 for 14 days improved overall well-being and relief vaginosis symptoms (Reid et al., 2001).

The meta-analysis of five studies conducted by Grin et al. questioned the effectiveness of *Lactobacillus* strains for the treatment and prophylaxis of RUTIs in adult women (Grin et al., 2013). The authors concluded that *Lactobacillus* strains prevent recurrent infections and are safe to use. A recent meta-analysis that evaluated the effectiveness of probiotics for prophylaxis of UTIs in premenopausal women concluded that probiotics do not prevent recurrent infections compared to placebo (Abdullatif et al., 2021). A potential difference in the result of these two meta-analyses was that Grin’s study excluded studies “using ineffective strains.” In contrast, Abdullatif’s study used a variety of *Lactobacillus* strains administered via the oral route and intravaginally. These meta-analyses collectively concluded that only a few strains of *Lactobacillus* could effectively treat or prevent RUTIs in women.

Unfortunately, little evidence exists about the effectiveness of probiotic treatments of UTIs or asymptomatic bacteriuria in pregnant women. Up to 15 percent of pregnant women would have asymptomatic bacteriuria, and ∼30% of them can develop cystitis and acute pyelonephritis (Kass 1970; Smaill and Vazquez 2019). About 2–10% of pregnant women carry GBS (Mead and Harris 1978; Matuszkiewicz-Rowinska et al., 2015; Szweda and Jozwik 2016; Abou Heidar et al., 2019). The GBS colonization may lead to intrauterine fetal death and neonatal sepsis (Wood and Dillon 1981). However, past GBS infections or inflammation induced by anti-GBS antibodies may be associated with premature labor (Møller et al., 1984; McKenzie et al., 1994). Therefore, screening for and eliminating GBS vaginal colonization is essential for preventing pregnancy complications. A randomized clinical study conducted in 2016 by Ho et al. investigated oral *Lactobacillus rhamnosus* GR-1 and *Lactobacillus reuteri* RC-14 treatment on vaginal colonization with GBS in pregnant women (Ho et al., 2016). About 43% of pregnant women previously positive for vaginal and rectal GBS taking oral probiotics were tested negative for GBS. It appears that this study has initiated another randomized clinical trial (Gutierrez 2017). This randomized clinical trial carried out by Gutierrez in 2017 had an intent to investigate the effectiveness of probiotics *Lactobacillus reuteri* DSM 16666/ATCC 55845 and *Lactobacillus reuteri* DSM 17938 for the treatment of bacteriuria and cystitis in pregnant women. The study’s primary outcomes were reducing symptoms and bacteriuria after 7-days treatment. Unfortunately, the results of this study were not published. An additional study conducted by Lui et al. investigated the effects of *Lactobacillus rhamnosus* GR-1 and *Lactobacillus reuteri* RC-14 on GBS levels in the vagina of women in the third trimester of pregnancy who had GBS- positive vaginal cultures (Liu et al., 2020). The authors also investigated the effects of probiotics on pregnancy outcomes. The probiotic treatment decreased the percent of women with GBS-positive cultures and premature rupture of membranes.

#### 3.4.2 Probiotic Treatment of Urinary Tract Infections in Children

A few clinical trials have investigated the effectiveness of various probiotics for treating UTIs in children with normal development of urinary tract and children with primary VUR. A small retrospective study investigated the effectiveness of therapy combining fluoroquinolone for 14 days and probiotic (*S. boulardii*) for 1 year in children with RUTIs (Madden-Fuentes et al., 2015). The study has shown a significant reduction in UTI episodes after treatment initiation. Seventy percent of children (7/10) were free of UTIs during the follow-up period (3–15 months).

The effectiveness of probiotic prophylaxis of RUTIs in infants with pyelonephritis was compared with antibiotic therapy or no-prophylaxis (Lee et al., 2016). Only 8.2% of infants had RUTIs during a 6-month follow-up period. However, probiotic therapy was more effective than no-prophylaxis; however, the effectiveness of probiotics was comparable to antibiotic treatment.

The vast majority of children with an anatomically normal urinary tract recovered from their first febrile UTI who were treated with a probiotic formulation containing *Lactobacillus acidophilus, Lactobacillus rhamnosus, Bifidobacterium bifidum, and Bifidobacterium lactis* and were subsequently free of UTI recurrence for 18 months (Sadeghi-bojd et al., 2019). A randomized, double-blind placebo-controlled clinical trial was initiated by Daniel et al. that investigated the effectiveness of probiotics *Lactobacillus rhamnosus* PL1 and *Lactobacillus plantarum* PM1 in preventing UTI in children (Daniel et al., 2020). The primary outcomes of this study were the frequency of RUTIs during the intervention and the follow-up period (9 months after the intervention). Unfortunately, no results were reported.

The ability of *Lactobacillus acidophilus* probiotic to prevent RUTIs in children with persistent primary VUR was compared to an antibiotic treatment (TMP-SMX) (Lee et al., 2007). It has been determined that both treatments could effectively prevent RUTIs in these patients. The effectiveness of a combinational therapy of probiotics *Lactobacillus acidophilus* and *Bifidobacterium lactis* with nitrofurantoin in preventing RUTIs was compared with nitrofurantoin alone in children with unilateral VUR and RUTIs (Mohseni et al., 2013). The combinational treatment reduced the number of RUTIs in treated patients. The difference between the probiotic plus antibiotic group and the antibiotic group was not statistically significant.

A meta-analysis of randomized clinical trials analyzing the effectiveness of probiotics in children with RUTIs concluded that probiotics are more effective than placebo, and the effectiveness of probiotics was comparable to antibiotic treatment (Meena et al., 2021). However, another meta-analysis has shown that probiotic therapy for treating RUTIs in children is effective only when combined with antibiotics (Hosseini et al., 2017).

The review of available evidence on the effectiveness of probiotics led us to conclude that the most effective in preventing RUTIs in pregnant women and children appear to be *Lactobacillus* strains. Altogether, probiotics can better prevent RUTIs than a placebo; they are comparable in effectiveness to antibiotic treatments. Potentially, a combination of probiotics with antibiotics may be most beneficial in preventing RUTIs.

### 3.5 Use of Multi-Component Formulations for the Treatment of Urinary Tract Infection

Many proprietary blends and phytodrugs are available on the market to treat and prevent UTIs in adults. In addition, two multi-component formulations have been identified, the effectiveness of which has been tested in pregnant women and children.

#### 3.5.1 Canephron® N

Canephron® N is a well-known registered multi-component herbal remedy containing centaury powder (*Centaurium erythraea* Rafn), lovage roots (*Levisticum officinale* Koch), and rosemary leaves (*Rosmarinus officinalis* Linné) (Naber 2013). According to a Bionorica leaflet information, the remedy (phytodrug) is recommended as a supportive therapy of conditions associated with “inflammatory diseases of the efferent urinary tract; to flush out the urinary tract in order to prevent the deposition of renal sand” in patients older than 12 years (Bionorica 2016).

Due to the presence of three medicinal herbs, the remedy demonstrates diuretic, anti-nociceptive, anti-spasmodic, and anti-adhesive effects (Höller et al., 2021). A retrospective analysis of the effectiveness of Canephron® N conducted by Höller et al. has concluded that Canephron® N treatment was associated with less sporadic RUTIs within 1 year after treatment compared to antibiotic therapies as well as fewer follow-up antibiotic prescriptions (Höller et al., 2021). The authors suggested that Canephron® N can treat UTIs and acute cystitis as an alternative symptomatic treatment. A phase III double-blind randomized clinical trial reported that 83% of women, age 18–70, with acute UTIs receiving Canephron® N had no additional antibiotic prescriptions between days 1 and 38 (Wagenlehner et al., 2018). Similar results were seen in the group receiving Fosfomycin. The effectiveness of Canephron® N treatment of UTIs was tested in pregnant women and children.

##### 3.5.1.1 Canephron® N Treatment of Urinary Tract Infection in Pregnant Women

The effectiveness of Canephron® N tablets and liquid drops was evaluated in a clinical study conducted by Potapov et al. in pregnant women with a range of UTIs (Potapo et al., 2004). While it is not clear whether this study was placebo-controlled or women in the study were randomized, the authors reported a significant reduction in pain, dysuria, nocturia, and pyuria. Another clinical trial investigated the efficacy of Canephron® N for the treatment of asymptomatic bacteriuria and chronic pyelonephritis (cohort 1) and chronic UTIs without exacerbation (cohort 2) in pregnant women (Ordzhonikidze et al., 2009). Pregnant women received two tablets three times a day for 3 weeks and then 1–2 weeks per month thereafter. Women suffering from UTI infections without exacerbations treated with Canephron® N (cohort) reported faster resolution of UTI symptoms than women in cohort 1. The authors concluded that Canephron® N had the most beneficial effects in women suffering from UTI infections without exacerbations.

One more study examined the efficacy of liquid form of Canephron® N for treating gestational pyelonephritis and chronic pyelonephritis with exacerbations in pregnant women with diabetes mellitus (Medved et al., 2015). The remedy (50 drops) was given to patients three times a day for 1 month. The authors reported a fast elimination of pyuria and a significant reduction in recurrences of pyelonephritis in treated women. One drawback of this study is that the results were compared to “historic” controls.

A retrospective analysis of data obtained following treatment of 28 pregnant women diagnosed with asymptomatic bacteriuria with Canephron® N was conducted by Lokshin (Lokshin 2018). The effectiveness of this remedy was compared with standard of care antibiotic therapy (*n* = 32). There were no statistically significant differences between the two groups in numbers of symptomatic UTIs and pyelonephritis cases. However, a few asymptomatic bacteriuria cases were registered in the Canephron® N group.

A few clinical studies investigated the effects of Canephron® N on the rates of congenital malformations of children born from mothers receiving the remedy during first, second, or third trimesters of pregnancy (Repina et al., 2006; Medved and Islamova 2009; Medved 2015). It appears that Canephron® N treatment in pregnant women did not result in any teratogenic, embryotoxic effects or defects of infant development.

##### 3.5.1.2 Canephron® N Treatment of Urinary Tract Infection in Children

A clinical study conducted by Voznesenskaya et al. investigated the effectiveness of Canephron® N for prophylaxis of recurrence of acute pyelonephritis in children (Voznesenskaya and Kutafina 2007). Children (*n* = 129, age 4 months–15 years old) were treated with liquid Canephron® N or nitrofurantoin for 3 months. While no recurrent infections were observed in both groups, Canephron® N was better tolerated by children than nitrofurantoin. A skin rash was reported in one child who received Canephron® N, while five children who received nitrofurantoin had nausea and vomiting, three children experienced constipation, and three children had a skin rash. Another clinical study investigated the effectiveness of treatment of UTIs in children (Sukalo et al., 2004). One group of children (*n* = 22, 5–17 years old) received Canephron® N in combination with antibiotic therapy and a second group received antibiotic therapy alone (*n* = 22, 5–17 years old). Only 20% of children receiving combinational therapy had “urinary syndrome” compared with 60% receiving antibiotics only when measured on day 15 after treatment initiation. While the authors did not specify improvement of which symptoms they were monitoring, we believe that the “urinary syndrome” may include the following: proteinuria, hematuria, leukocyturia, and potentially other signs.

Dilin et al. investigated the effectiveness of Canephron® N for treating dysmetabolic nephropathy with oxalate-calcium crystallization in children (Dilin et al., 2008). Children (2.7 months -17 years old) were treated with Canephron® N (*n* = 25) or a multivitamin formulation (vitamins A, E, and B6, *n* = 25) for 3 months. The treatment with Canephron® N resulted in a more rapid decrease of hematuria, calcinuria, oxaluria, and lipidemia. Lastly, Kirillov and others investigated the ability of Canephron® N to prevent urinary syndrome and RUTIs in children undergoing surgical correction of grade III-IV VUR (Kirillov et al., 2007). Among children who received Canephron® N, 86.4% had no recurrent infections compared to 77.3% of children who underwent surgery only. Urinary syndrome normalized in the Canephron® N group within 16.9 days and in the surgery group within 26.4 days after treatment initiation.

The analysis of presented clinical trials suggests that Canephron® N effectiveness is comparable to antibiotic effectiveness in children and pregnant women experiencing asymptomatic bacteriuria and UTIs and can improve antibiotic therapy when administered concurrently. It appears that Canephron® N is mainly effective in the prophylaxis of RUTIs.

#### 3.5.2 Cystenium II

Cystenium II (aka, Cistenium II) tablets/lozenges are dietary supplements intended to support adults’ and children’s urinary tract health (Vidal 2021). The supplement contains cranberry fruit extract standardized by proanthocyanidins A, D-mannose, and vitamin C. Cranberry-driven polyphenols may prevent UTI-causing bacteria from adhering to epithelial cells of the urinary tract (González de Llano et al., 2020). D-mannose prevents bacteria from binding to epithelial cells by attaching to bacterial type 1 pili, specifically adhesin FimH (Bouckaert et al., 2005). D-mannose may also act as an immunomodulator stimulating TGF-β production and regulatory T cell differentiation (Zhang et al., 2017).

Cystenium II is recommended for children older than 7 years of age to consume one lozenge twice per day for 2 weeks (Vidal, 2021). Pregnant women should take this remedy under physician supervision. A clinical trial evaluated the effectiveness of Cystenium II for the treatment of asymptomatic bacteriuria, acute cystitis, or exacerbation of chronic cystitis in pregnant women (Nashivochnikova and Leanovich 2020). One group of women received fosfomycin trometamol (3 g/day/14 days) and Cystenium II (1 lozenge/day). Another group was treated with fosfomycin trometamol only. The combined treatment with fosfomycin trometamol and Cystenium II effectively eliminated bacteria from the urinary tract in ≥70% of treated women by day 14 of treatment, and monotherapy with fosfomycin trometamol led to the elimination of pathogenic bacteria in 61% of women. Overall clinical effectiveness, measured by recurrency of asymptomatic bacteriuria or exacerbation of chronic cystitis in the combined therapy group, was 82–90%, and in the monotherapy group was only 56.5%.

We could not find any published clinical trial data investigating the effectiveness of this dietary supplement in children. However, Kotov and Petrov, in their article discussing the prophylaxis of RUTIs, stated that based on the safety profile of herbal remedies, Cystenium II can be used for the treatment of lower UTIs in children 7 years of age and older (Kotov and Perov 2020).

## 4 Discussion

Cephalosporins, penicillins, and nitrofurantoin antibiotics are commonly used to treat UTIs in pregnant women, while cephalosporins, TMP-SMX, and nitrofurantoin antibiotics are typically used to treat UTIs in children (Figures 1, 2). It has been reported that mono-antibiotic therapy for uncomplicated UTIs may lead to the development of resistance to antibiotics (Ears-Net 2019). About 50% of *E. coli* and >30% of *K. pneumoniae* clinical isolates were resistant to at least one antibiotic recommended for treatment of UTI in the European Union. Several genes have been identified in microorganisms causing UTIs that made bacteria resistant to carbapenems, ciprofloxacin and potentially other antimicrobial agents (Rajivgandhi et al., 2018a; Rajivgandhi et al., 2021). The treatment of uncomplicated UTIs with two or more antimicrobial agents appears to significantly decrease the number of antibiotic-resistant isolates (Zyryanov et al., 2020). However, the problems associated with acquiring resistance to antimicrobial therapy still exist and are a reason for concern. Additionally, many gram-negative bacteria that serve as the causative agents of UTIs are capable of forming biofilms that make bacteria less accessible to antibiotics. Some novel compounds have shown the ability to prevent biofilm formation by gram-negative bacteria (Rajivgandhi et al., 2018b; Rajivgandhi et al., 2018c; Rajivgandhi et al., 2019).

The analysis of existing clinical studies evaluating the effectiveness of natural therapies like cranberry extracts, HA, ascorbic acid, and probiotics for treating UTIs in pregnant women and children demonstrated that concomitant administration of natural productswith antimicrobial drugs enhances the efficacy of conventional treatments (Figures 1, 2). This is evidenced by the improved outcomes and faster recovery of patients when conventional drugs are administered in combination with natural products. In addition, the supplementation with cranberry, HA, ascorbic acid, and particular strains of probiotics appear to result in decreased RUTIs and may gain the potential to decrease antimicrobial resistance in pregnant women and children.

The effectiveness of other natural therapies has been investigated occasionally in children but primarily in adult patients. These include phytodrug Eucalimin, multi-component herbal remedies Brusniver and Elecasol, a supplement known as Uronext®, and D-mannose.

Eucalimin is a sum of triterpene phenol aldehyde and triterpenoid compounds isolated from foliage and shoots of *Eucalyptus Viminalis* Labill (Enioutina et al., 2017). This phytodrug demonstrates effectiveness against gram-negative bacteria, including clinical isolates resistant to conventional antibiotics (Enioutina et al., 2017). A clinical trial investigated the effectiveness of Eucalimin suppositories in 40 children (4–14 years of age) with cystitis and vulvovaginitis (Vichkanova and Krutikova 2012). Eucalimin treatment eradicated bacteriuria and leukocyturia in these patients. Symptoms of the disease disappeared in children treated with Eucalimin much faster than in children treated with standard of care drugs (e.g., cephalosporins).

Limited clinical evidence of the D-mannose effectiveness in pregnant women and children exists. A clinical trial compared D-mannose effectiveness to nitrofurantoin for prophylaxis of UTIs in women. The study revealed that D-mannose significantly reduced the risk of RUTIs and was not different from nitrofurantoin prophylaxis of UTIs (Kranjcec et al., 2014). In addition, in a small study (11 pediatric patients) that investigated the prophylactic administration of D-mannose in “complex paediatric urology patients,” D-mannose appeared to reduce the risk of UTIs by 53% (Brownlee et al., 2020).

Two multi-component formulations, Canephron® N and Cystenium II, reviewed in this manuscript, appeared to effectively treat UTIs in pregnant women and children when used in combination with standard of care medicines. The data suggest the Canephron® N treatment may be used as monotherapy to treat UTIs in pregnant women, and its’ effectiveness is comparable to antibiotic therapy. Cystenium II is potentially effective as a single complementary therapeutic in pregnant women but needs to be evaluated in children. Dietary supplements with similar ingredients are available on the market (e.g., Uronext®). This supplement contains D-mannose, cranberry extract, and vitamin D. It is recommended to support the urinary tract of adults and pregnant women after consultations with a physician (Uronext®, 2020).

Brusniver and Elecasol, multi-component formulations, can be potentially used as standalone therapies or as complementary treatments to antibiotics. Brusniver is a mixture of foliage of *Vaccinium vitis-idaea* L (50%), the aerial part of *Hypericum perforatum* L. and *Hypericum maculatum* Crantz (*H*. Quadrangulum L.) (20%), fruits of *Rosa* L. (20%), the aerial part of *Bidens tripartite* L. (10%) (Vichkanova et al., 2004). This phytodrug has antimicrobial properties against *E. coli*, *Pseudomonas aeruginosa,* and *Proteus* and demonstrates anti-inflammatory and diuretic properties. The effectiveness of Brusniver was tested in 31 patients with cystitis, pyelonephritis, and other UTIs (Vichkanova et al., 2004). Brusniver demonstrated a good safety profile; treated patients did not report any adverse reactions. More than 83% of treated patients had positive outcomes. Elecasol is another phytodrug comprised of a mixture of *Eucalyptus viminalis* Labill. (20%), *Salvia officinali*s L. (20%); *Glycyrrhiza geabra* L. and *Glycyrrhiza uralensis* Fisch (20%), *Calendula officinalis L.* (20%), *Matricaria reculita* L and *Matricaria Chamomilla* L) (10%), *Bidens tripartite* L. (10%) (Vichkanova et al., 2009). This phytodrug has both anti-microbial and anti-inflammatory properties. The effectiveness of Elecasol has been tested in adult patients with chronic pyelonephritis, cystitis, and prostatitis (Vichkanova et al., 2009). Patients received Elecasol as monotherapy or in combination with antibiotics. Ninety percent of patients with pyelonephritis receiving antibiotics followed by the Elecasol treatment demonstrated long-lasting remission.

We also would like to mention a long-time discovered agent which has unfortunately been overshaded by antibiotics known as bacteriophages viruses infecting bacteria. Bacteriophages can represent a good clinical approach for the therapy of UTIs caused by antibiotic-resistant microorganisms (Kortright et al., 2019). Other potential natural compounds that can be used for the treatment of UTIs include natural antimicrobials peptides produced by a variety of organisms (Lei et al., 2019). Some of these antimicrobial peptides are defensins, cathelicidins, and antimicrobial peptide drugs approved by the FDA (e.g., Daptomycin and Telaprevir). Unfortunately, the naturally occurring peptides have a short half-life and may exhibit toxicity, including severe hemolytic activity (Lei et al., 2019). Lectin antagonists could be of interest to researchers as alternative or complementary treatments for multi-drug-resistant infections, including UTIs (Meiers et al., 2019). Lectins are proteins that are used by microorganisms to infect host cells and maintain infection. The naturally occurring lectin ligands are bacterial peptidoglycans or lipopolysaccharides. It appears that the use of lipopolysaccharides is quite problematic due to severe adverse reactions to the bacterial products. However, monovalent biofilm targeting glycomimetics may be used in a combination with conventional drugs or as a stand-alone therapy. Exploring additional phytodrugs, multi-component herbal remedies, and natural products in pregnant women and children would provide added therapeutic options for these special populations.

## 5 Conclusion

The administration of antimicrobial drugs is the current standard of treatment for UTIs in pregnant women and children. Antibiotics can effectively treat UTIs in both patient populations; however, the prescription of antibiotics prophylactically raises questions of their effectiveness in preventing RUTI. Additionally, the long-term use of antibiotics and difficulties in dosing antibiotics in young children may lead to the growing number of antibiotic-resistant bacteria.

The addition of natural products like cranberry, HA, ascorbic acid, probiotics, Canephron® N, and Cystenium II to conventional treatments of acute UTIs or as a prophylactic regimen for treatment RUTIs can benefit both pregnant women and children. However, it appears that some natural therapies can be used as complementary medicine to antibiotic treatment and as monotherapy of UTIs in both populations. Additional exploration of the potential of new natural medicines as monotherapy or when used in combination with antimicrobial drugs in pregnant women and children with UTIs is warranted and would provide added therapeutic options. Overall, there appears to be a benefit in using natural products, but limited information remains available on the safety of many of these natural products. We conclude that based on our review, historical information, and clinicians’ experience in using these natural remedies for treating UTIs, there are minimal adverse effects reported, and they seem safe and well-tolerated by pregnant women and children.

## Glossary

CUTIE: Careful Urinary Tract Infection Evaluation
CDC: Center for Disease Control and Prevention
CFU/ml: Colony-forming units per milliliter
GBS -: Group B *Streptococcus*
IDSA -: Infectious Diseases Society of America
NB -: neurogenic bladder
PRIVENT -: Prevention of Recurrent Urinary Tract Infection in Children with VUR and Normal Renal Tracts
RIVUR -: Randomized Intervention for Children with Vesicoureteral Reflux
RUTIs -: recurrent UTIs
TMP-SMX -: trimethoprim-sulfamethoxazole
UTI-: urinary tract infection
VUR -: vesicoureteral reflux
